# Analysis of ROH Characteristics Across Generations in Grassland-Thoroughbred Horses and Identification of Loci Associated with Athletic Traits

**DOI:** 10.3390/ani15142068

**Published:** 2025-07-13

**Authors:** Wenqi Ding, Wendian Gong, Tugeqin Bou, Lin Shi, Yanan Lin, Xiaoyuan Shi, Zheng Li, Huize Wu, Manglai Dugarjaviin, Dongyi Bai

**Affiliations:** 1 Key Laboratory of Equus Germplasm Innovation (Co-Construction by Ministry and Province), Ministry of Agriculture and Rural Affairs, Hohhot 010018, China; dingwenqi0331@gmail.com (W.D.); gongwendian1996@outlook.com (W.G.); tvgqin@gmail.com (T.B.); 19832607527@163.com (L.S.); linyanan@emails.imau.edu.cn (Y.L.); xiaoyuans2021@163.com (X.S.); lzheng0511@sina.com (Z.L.); whz020419@163.com (H.W.); dmanglai@163.com (M.D.); 2 Inner Mongolia Key Laboratory of Equine Science Research and Technology Innovation, Inner Mongolia Agricultural University, Hohhot 010018, China; 3 Equus Research Center, College of Animal Science, Inner Mongolia Agricultural University, Hohhot 010018, China

**Keywords:** Grassland-Thoroughbreds, whole-genome resequencing, ROH

## Abstract

The core objective of racehorse breeding is to enhance the speed and endurance of the horses. The Grassland-Thoroughbred is an emerging horse breed developed in northern China in recent years, characterized by excellent speed performance, enduring stamina, and strong environmental adaptability. However, research on the genetic characteristics within this breed and the genes associated with athletic performance remains relatively limited. We conducted whole-genome resequencing of Grassland-Thoroughbred F1, F2, F3, and the crossbred population (CY) and obtained a total of 4056.23 Gb of high-quality data after quality control. The single nucleotide polymorphisms (SNPs) were primarily distributed in intergenic regions, followed by intronic regions. Principal component analysis (PCA) and STRUCTURE revealed clear distinctions among the generations, with a notable overlap between CY and F3. Using the SNP dataset, we analyzed the number and length distribution patterns of runs of homozygosity (ROHs) in the genomes of different generational groups of Grassland-Thoroughbreds. Short ROHs ranging from 0.5 to 2 Mb were the most abundant, with the following distribution: F1 (85.15%) > F2 (82.92%) > CY (78.75%) > F3 (77.51%). Medium-length ROHs (2–8 Mb) and long ROHs (>8 Mb) together exhibited a similar but opposite trend. The average length of ROHs was 1.57 Mb. The inbreeding coefficients (F_ROH) among different generational groups of Grassland-Thoroughbreds were as follows: F1 (0.0942) < F2 (0.1197) < CY (0.1435) < F3 (0.1497). Through ROH island analysis, 10 high-frequency ROH regions were identified and annotated with 120 genes. Genomic regions and candidate genes associated with athletic traits—*ACAD8*, *OPCML*, *PRDX2*, *NTM*, *NDUFB7*, *SCL25A15*, *FOXO1*, and *SLC4A10*—were identified. These genes may play important roles in regulating muscle performance, mitochondrial energy supply, and learning and memory processes in horses and are closely associated with the athletic ability of the Grassland-Thoroughbred population. This study is the first to systematically characterize the genomic diversity and inbreeding dynamics of the Grassland-Thoroughbred during the breeding process. It identifies candidate genes that may influence athletic performance, thereby providing an important molecular foundation and theoretical basis for the genetic improvement and performance-based selection of this emerging breed.

## Simple Summary

The Grassland-Thoroughbred is an excellent racehorse breed developed in northern China in recent years, characterized by speed, endurance, and environmental adaptability. In this study, whole-genome resequencing was conducted on different generational groups of this breed to systematically analyze the distribution of runs of homozygosity (ROHs), population structure, and inbreeding levels. A series of candidate genes related to athletic performance, such as *FOXO1*, *PRDX2*, and *SLC25A15*, was identified. The findings provide molecular insights into the genetic basis of this breed and offer valuable guidance for optimizing breeding strategies, thereby promoting its development and application in the racing industry.

## 1. Introduction

Since ancient times, horses have held a significant role in human society. The Mongolian horse, one of China’s oldest native breeds, is internationally recognized as an outstanding local breed. It is predominantly found throughout the Inner Mongolia Autonomous Region and neighboring areas [1]. Renowned for its remarkable endurance, broad adaptability, and strong resilience, the Mongolian horse is frequently used as the maternal line in crossbreeding programs. The Xilingol horse was developed over 35 years of systematic breeding, with the Mongolian horse serving as the maternal parent, thereby inheriting many of its superior traits [2]. Thoroughbreds, recognized as the premier breed in modern horse racing, particularly excel in sprint events. Owing to their exceptional genetic qualities, they are frequently used to improve the speed and endurance of other horse breeds [3,4]. With societal progress, the roles of horses have continually evolved. Since 1995, aiming to further improve horses’ athletic performance, physique, and speed, Inner Mongolia has introduced Thoroughbred and Xilingol horses for crossbreeding. After nearly three decades of continuous selective breeding and performance evaluation, a new breed combining both speed and endurance has been developed. It has not yet received official national breed recognition and is provisionally named the Grassland-Thoroughbred. These horses are affectionately known as “Sunflower Horses” due to the distinctive sunflower-shaped brand on their hips. Currently, there are at least 2500 registered individuals [5]. The breeding goals mainly focus on improving the horses’ speed performance while enhancing their adaptability to the harsh grassland environment, in order to meet the demands of modern horse racing and related industries. Relevant management organizations have established preliminary breed registration and pedigree management systems. The development of native racing horse breeds in China not only enhances the competitiveness of the domestic horse racing industry but also contributes significantly to regional economic and cultural growth [6].

Compared to traditional pedigree analysis methods, such as kinship analysis and lineage tracing, modern genomic technologies provide deeper insights into genetic diversity, population history, and selected traits [7,8]. Among these, runs of homozygosity (ROHs) serve as a crucial genomic tool, defined as continuous, extensive homozygous segments in the genome resulting from an individual inheriting identical haplotypes from both parents. This measure has been widely employed to evaluate the loss of genetic diversity within breeds [9]. The length and number of ROHs reflect an individual’s inbreeding level and the population’s historical background. Longer ROHs typically indicate recent inbreeding events, whereas shorter ROHs may originate from ancient population bottlenecks or ancestral inheritance. Currently, ROH is a key indicator for estimating genomic inbreeding levels (F_ROH) [10]. Unlike pedigree-based inbreeding coefficients, F_ROH can capture variation arising from Mendelian segregation and genetic linkage [8], helping to assess the maintenance of genetic diversity and potential genetic risks during the breeding process. The distribution patterns of ROHs can reflect the genetic structure and selection signatures of populations. Genomic regions exhibiting the highest frequency of ROHs within a population are termed “ROH islands” [11]. These ROH islands are typically population-specific, and genes showing selection signatures often coincide with these regions [12]. As such, ROH islands are extensively used to identify potential selective genomic regions and play a vital role in studying adaptive evolution within particular populations [9]. For example, Sievers J evaluated the genetic diversity of the Rhenish German Coldblood horse population using both pedigree and genomic data. The inbreeding levels calculated from pedigree and ROHs showed a moderate correlation. Due to the decline in genetic diversity, the population size has decreased significantly. ROH island analysis identified genes associated with reproduction and morphological traits [13]. In the Polo Argentino breed, genomic ROH analysis of 506 horses revealed a moderate level of ancestral inbreeding and identified ROH islands associated with behavior, neurodevelopment, and metabolic functions, providing new insights into the genetic basis and breeding strategies of polo horses [14]. In recent years, with the advancement of high-throughput sequencing technologies, ROH analysis has become an important tool for evaluating the genetic structure of horse populations and improving genetic resources.

Based on whole-genome data from the F1, F2, and F3 generations and crossbred populations of Grassland-Thoroughbreds, this study explored the genetic characteristics and their evolutionary trends across generations and evaluated the extent of inbreeding. In addition, it identified potential functional loci and candidate genes associated with athletic performance in Grassland-Thoroughbreds. This work constitutes the first whole-genome analysis within this population. The results offer a theoretical foundation and valuable data support for genetic improvement and marker-assisted selection in this breed, thereby establishing a solid basis for the formulation of scientifically grounded breeding strategies.

## 2. Materials and Methods

### 2.1. Sample Collection

A total of 139 horses were included in this study, comprising 104 samples (F1 = 46, F2 = 28, F3 = 30) newly collected for this study and 35 previously published Grassland-Thoroughbred samples (crossbred generation). The Grassland-Thoroughbred samples were obtained from the Inner Mongolia Grassland-Thoroughbred Breeding Co., Ltd., (Inner Mongolia, China) which maintains detailed pedigree records and a structured breeding program for this population (Supplementary Figure S1). According to pedigree records, individuals with close genetic relationships were excluded. The sample collection in this study was approved by the Animal Ethics Committee of Inner Mongolia Agricultural University (No: NND2023085). All procedures were conducted in accordance with the Regulations on the Administration of Laboratory Animals and relevant international guidelines. All blood samples were collected from the jugular vein of each horse by trained professionals, placed in EDTA anticoagulant tubes, aliquoted, rapidly frozen in liquid nitrogen, and subsequently stored at −80 °C to ensure sample quality for subsequent experiments.

### 2.2. Library Construction

Genomic DNA was extracted from the collected blood samples using the CTAB method. The average DNA concentration was measured at 103.58 ng/µL using the Agilent 5400 system (Agilent Technologies, Santa Clara, CA, USA) (Supplementary Table S1). For each sample, 1 µg of genomic DNA was used to construct sequencing libraries. Library preparation was performed with the NEBNext^®^ Ultra™ DNA Library Prep Kit (New England Biolabs, Ipswich, MA, USA) for Illumina, following the manufacturer’s protocol. Genomic DNA was fragmented to an average size of approximately 350 bp via ultrasonication. The resulting fragments were then subjected to end repair, A-tailing, and ligation with Illumina sequencing adapters. Subsequently, PCR amplification was carried out to enrich the adapter-ligated DNA fragments. The amplified libraries were purified using the AMPure XP system (Beckman Coulter, Brea, CA, USA). The average DNA concentration was measured using a Qubit fluorometer, and DNA integrity was assessed using the Agilent 5400 TapeStation. Libraries meeting the required concentration and quality standards were submitted to Novogene Bioinformatics Technology Co., Ltd. (Beijing, China) for high-throughput sequencing. Sequencing was performed on the Illumina HiSeq 4000 platform (San Diego, CA, USA)using a paired-end 150 bp strategy, achieving an average sequencing depth of 10× per sample.

### 2.3. Quality Control and Data Filtering

We employed fastp (version 0.23.1) to conduct quality control on the raw sequencing data, ensuring the reliability of subsequent analyses. The quality control criteria were as follows: (1) reads containing ≥5% unidentified bases (N) were discarded; (2) reads with more than 20% of bases having a Phred quality score below 15 were removed; (3) reads containing adapter sequences were filtered out, allowing up to 3% mismatches; and (4) reads shorter than 15 bases were excluded [15]. Subsequently, the processed data were assessed using FastQC (version 0.11.5) to evaluate data quality and confirm their suitability [16].

### 2.4. Variant Detection and Annotation

The clean data were aligned to the horse reference genome EquCab3.0 using BWA (version 0.7.15) [17]. The resulting alignment files were subsequently converted and sorted using Samtools (version 1.3). To eliminate potential PCR duplicates, Picard tools were applied with the MarkDuplicates command, setting REMOVE_DUPLICATES = true. SNP calling was then performed on the recalibrated BAM files using GATK HaplotypeCaller (version 4.0.3.0), followed by merging the data with CombineGVCFs. The SelectVariants module was used to extract raw SNPs and raw indels. SNP filtering was conducted using the VariantFiltration function with the following parameters: QUAL < 30.0, QualByDepth (QD) < 1.5, RMS Mapping Quality (MQ) ≥ 4, and Depth of Coverage (DP) < 5. After filtering, a VCF file containing high-quality SNPs was generated. Additional filtering was carried out using VCFtools as follows: SNPs missing in more than 80% of samples were removed (--max-missing 0.8), variants with a minor allele frequency (MAF) greater than 0.05 were retained (--maf 0.05), only biallelic variants were kept (--min-alleles 2 --max-alleles 2), and loci with a quality score (QUAL) ≥ 30 were preserved. To ensure data accuracy and consistency, SNPs not mapped to autosomes or located on sex chromosomes were excluded [18].

### 2.5. Population Structure Analysis

Prior to analysis, linkage disequilibrium pruning was performed using PLINK (version 1.9) with the indep-pairwise command. The parameters were set to a window size of 50 SNPs, a step size of 5 SNPs, and an r^2^ threshold of 0.2 to retain a set of independent SNP markers [19]. Principal component analysis was employed to explore genetic variation and phenotypic differentiation among individuals. PCA was conducted on all horses using PLINK (version 1.9), with the parameter --pca 10, to extract the top two principal components that account for the majority of the variation in the SNP dataset. These two principal components were subsequently visualized using the ggplot2 package (version 3.3.5) in R (version 3.6.1) to investigate clustering patterns across different horse populations [20]. To further evaluate the genetic structure of the populations and detect potential population stratification, the filtered VCF file was converted into the BED format required by ADMIXTURE (version 1.3.0) using PLINK. ADMIXTURE determines the optimal number of ancestral populations based on the best K value. The final population structure results were visualized using TBtools (version 2.154).

### 2.6. ROH Analysis

Runs of homozygosity (ROHs) for each individual were identified using the --homozyg function in PLINK with the following parameter settings: (1) a minimum ROH length of 50 kb; (2) a maximum of 5 missing genotypes and up to 3 heterozygous genotypes allowed per segment; (3) a requirement of at least 50 consecutive SNPs; (4) a minimum SNP density of 0.05 SNPs per kb; and (5) a maximum gap of 1 Mb between adjacent homozygous SNPs. These parameters help avoid the identification of short ROH segments caused by genotyping errors or random homozygosity, ensuring that ROH segments cover a sufficient number of SNPs, thereby improving the reliability and accuracy of the identification. To enable a more detailed analysis of ROH patterns across generations, the ROH segments were classified into six length categories: 0.5–1, 1–2, 2–4, 4–8, 8–16, and >16 Mb. Based on the length of ROH segments, they can be classified into three categories: short ROHs (0.5–2 Mb), medium ROHs (2–8 Mb), and long ROHs (>8 Mb). The length of ROHs is closely related to the timing of inbreeding events: short ROHs reflect ancient inbreeding or population bottlenecks, while long ROHs indicate recent inbreeding events. Data visualization was performed using the ggplot2 package in R to clearly illustrate the distribution patterns of ROHs across different generations.

The inbreeding coefficient F_ROH is a key parameter for evaluating the level of inbreeding within a population. It is calculated using the formula: F_ROH = ∑L_ROH_/Lauto, where ∑L_ROH_ denotes the total length of ROH segments within an individual’s genome, and Lauto represents the length of the autosomal genome covered by SNPs in the whole-genome sequencing data.

The proportion of SNPs located within ROHs was estimated by calculating how frequently each SNP appeared in ROH segments and dividing this by the total number of individuals. To identify genomic regions with high levels of homozygosity—referred to as ROH islands—the top 0.1% of SNPs based on the homozygosity rate distribution were selected as candidate regions. Genes located within these ROH islands were annotated using the EquCab3.0 genome annotation file obtained from the NCBI database. The biological functions of these genes were subsequently summarized through a comprehensive literature review.

### 2.7. Functional Enrichment Analysis

Functional enrichment analysis enables in-depth exploration of the specific type of equine traits associated with performance or health traits in horses. Using the online tool DAVID, Gene Ontology (GO) and Kyoto Encyclopedia of Genes and Genomes (KEGG) enrichment analyses were performed on the candidate genes. To ensure the biological relevance of the enrichment results, only annotation terms with a *p* < 0.05 were retained. To explore the functional relationships among ROH candidate genes, protein–protein interaction (PPI) networks were constructed using the STRING database with Equus caballus as the reference species. The interaction data were imported into Cytoscape (version 3.7.1) for network visualization and further analysis.

## 3. Results

### 3.1. Sequencing Data and Genome-Wide Genetic Variation

This study included a total of 104 Grassland-Thoroughbred horses from three hybrid generations (F1, F2, F3), along with 35 previously published NGS datasets of the Grassland-Thoroughbred crossbred population (CY). A total of 4056.23 Gb of clean data was generated, with an average sequencing depth of 12.32× (Supplementary Table S2). The majority of SNPs were located in intergenic regions, followed by intronic regions, while a smaller proportion was distributed in exonic regions, splice sites, and upstream and downstream regions of genes (Supplementary Table S3). The number of SNPs on each chromosome was positively correlated with chromosome length, with chromosome 1 (Chr1) being the longest chromosome and containing the highest number of SNPs, whereas Chr31, the shortest chromosome, had the fewest SNPs (Supplementary Figure S2).

### 3.2. Population Structure Analysis

**Figure 1:**
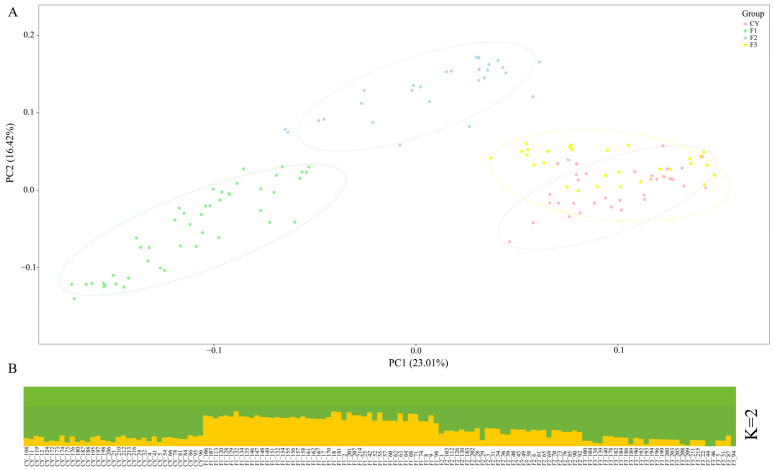
Genetic structure analysis of different generations of the Grassland-Thoroughbred population. (**A**) Principal component analysis (PCA), F1: First-generation hybrids, F2: Second-generation hybrids, F3: Third-generation hybrids, CY: Grassland-Thoroughbred (crossbred); (**B**) Population structure analysis (different colors represent different ancestral components).

To characterize the genetic structure of the Grassland-Thoroughbred populations across generations, PCA was performed on the autosomal SNP datasets of F1, F2, F3, and CY (Figure 1A). The results showed that the first principal component (PC1) clearly distinguished the generational groups, explaining 23.01% of the total genetic variation, indicating significant genetic differences among generations. However, a notable overlap was observed between the F3 generation and the CY population, which may be due to backcrossing that did not significantly alter allele frequencies, reflecting a high degree of genetic similarity between the two groups. ADMIXTURE further supported this observation. When the number of ancestral populations was assumed to be K = 2, the genetic structure was divided into two ancestral components: Thoroughbred and Xilingol horse. Individuals of the F1 generation exhibited approximately a 1:1 ancestral proportion, consistent with the genetic expectation of first-generation hybrids. With continued backcrossing to Thoroughbreds, the genetic composition of the F2 and F3 generations gradually shifted toward the Thoroughbred lineage, with a corresponding decrease in the Xilingol horse component, in line with genetic principles. The CY population showed a high degree of genetic similarity to the F3 generation (Figure 1B), further confirming this trend.

### 3.3. ROH Patterns

**Figure 2:**
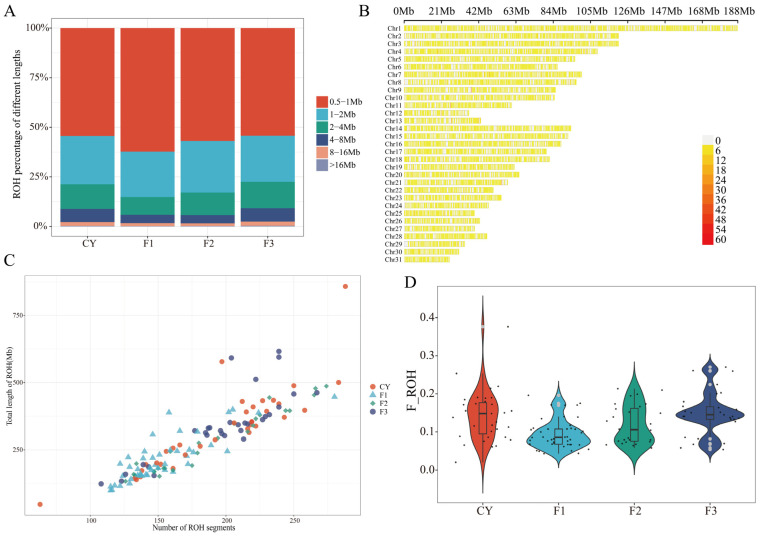
ROH patterns and inbreeding coefficients in horse populations across generations. (**A**) Proportion of different lengths of ROHs in horse populations across generations, where different colors represent the proportion of ROHs; (**B**) Distribution of the number of ROHs across different chromosomes; (**C**) Distribution of total length and number of ROHs in individuals across different generations of horse populations, where different colors represent individuals from different generational groups. The *y*-axis indicates the size of the genome covered, and the *x*-axis represents the number of ROH segments; (**D**) Violin plot of the genome inbreeding coefficients across horse populations in different generations.

In this study, a total of 139 samples were analyzed, yielding 25,036 runs of homozygosity (ROHs) of various lengths. Six distinct ROH length categories were statistically summarized (Figure 2A), with short ROHs ranging from 0.5 to 2 Mb being the most prevalent. The F1 generation exhibited the highest proportion of these short ROHs (85.15%), followed by F2 (82.92%), CY (78.75%), and F3 (77.51%). The combined number of medium-length ROHs (2–8 Mb) and long ROHs (>8 Mb) was greatest in the F3 generation (22.49%), followed by CY (21.25%). The average ROH length across all samples was 1.57 Mb. The distribution of ROHs across different Chrs was uneven. In general, the length of ROHs tended to increase with Chr length. Chr1 exhibited the highest number of ROHs (number = 2177), while Chr31 had the fewest (n = 232) (Figure 2B). ROH coverage was lowest on Chr12 and highest on Chr30. The scatter plot illustrating the distribution of ROH lengths and counts across different generations of Grassland-Thoroughbred horses (Figure 2C) shows substantial variation among individuals in both the number of ROHs and the total length of genome coverage. The average number of ROHs per individual varied significantly across generations (F1: 153, F2: 187, F3: 197, CY: 196), and the average total ROH length per individual in each generation followed a similar trend (F1: 1.37 Mb, F2: 1.41 Mb, F3: 1.68 Mb, CY: 1.6 Mb). As the number of ROHs increased, the cumulative ROH length per individual also increased accordingly. The total ROH length of an individual reflects the degree of homozygosity in its genome and can therefore be used to infer the level of inbreeding. The average inbreeding coefficient based on ROHs (F_ROH) in the Grassland-Thoroughbred breeding population was 0.124. Among the generations, the F3 generation exhibited the highest average F_ROH (0.149666), followed by CY (0.1435), F2 (0.119657), and F1, which had the lowest F_ROH (0.09428) (Figure 2D). By ANOVA, the differences in F_ROH among generations were significant (*p* = 1.88 × 10^−5^).

### 3.4. ROH Islands

**Figure 3:**
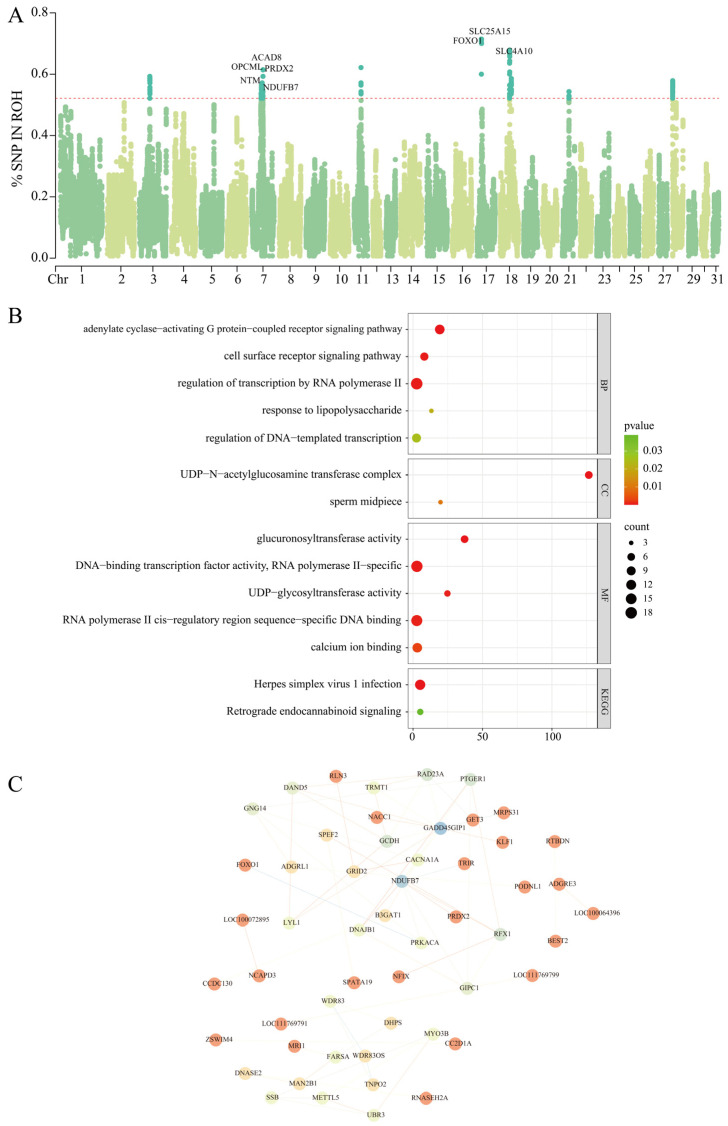
Identification and functional analysis of ROH islands in horse populations. (**A**) Manhattan plot of SNP percentage in ROHs; (**B**) Functional enrichment of candidate genes in ROH islands; (C) Functional interaction network of ROH-associated genes.

As nucleotide diversity decreases and homozygosity increases, certain genomic regions are more likely than others to form ROH islands. These ROH islands are not randomly distributed across the genome; rather, they are specific regions shared among all individuals in the population. To identify ROH-associated genomic regions across all individuals, according to the existing literature, the top 0.1% most frequent SNPs within ROHs (present in at least 50% of the samples) were considered candidate SNPs [21]. A total of 10 such regions were identified: one region on Chr 3 (46.68 Mb–47.35 Mb); three regions on Chr 7 (41.51 Mb–47.14 Mb, 48.28 Mb–48.88 Mb, and 50.44 Mb–50.45 Mb); one region on Chr 11 (31.31 Mb–31.55 Mb); one region on Chr 17 (18.31 Mb–18.94 Mb); two regions on Chr 18 (41.67 Mb–42.42 Mb and 49.35 Mb–50.14 Mb); one region on Chr 21 (29.89 Mb–30.51 Mb); and one region on Chr 28 (0.06 Mb–0.69 Mb) (Figure 3A). The number of SNPs detected within each of the 10 ROH islands ranged from 4 to 7254. These regions were subjected to gene annotation, with Chr 7 containing the highest number of annotated genes and Chr 3 the fewest. In total, 120 protein-coding genes were identified (Table 1). We then performed functional annotation of these candidate genes using GO and KEGG enrichment analyses, along with publicly available databases such as NCBI and Ensembl, to gain preliminary insights into their potential biological functions. Based on this analysis and a review of the relevant literature, eight genes were identified as being associated with athletic performance traits: *ACAD8*, *OPCML*, *PRDX2*, *NTM*, *NDUFB7*, *SLC25A15*, *FOXO1*, and *SLC4A10*.

### 3.5. Functional Enrichment

Through enrichment analysis of genes covered by high-frequency ROH segments, a total of 13 GO terms and 2 KEGG pathways were significantly enriched (*p* < 0.05) (Figure 3B) (Supplementary Table S4). The GO term analysis revealed that *FOXO1* and *PRKACA* were significantly enriched in positive regulation of gluconeogenesis (GO:0045722), adenylate cyclase-activating G protein-coupled receptor signaling pathway (GO:0007189), and regulation of transcription from RNA polymerase II promoter (GO:0006357). The gluconeogenesis process can promote glucose synthesis, providing energy support for horses during exercise. The KEGG enrichment results indicated enrichment in the Herpes simplex virus 1 infection pathway (ecb05168) and the retrograde endocannabinoid signaling pathway (ecb04723), which are mainly associated with disease and neural regulation. These ROH segments, present across different generations of the Grassland-Thoroughbred population, may have potential impacts on athletic performance, immune function, and environmental adaptability.

The constructed PPI network revealed several gene modules with varying degrees of connectivity (Figure 3C), suggesting potential cooperative regulatory functions among these genes. Notably, genes such as *FOXO1*, *PRDX2*, and *NDUFB7* were located at the central positions of the network and exhibited high connectivity, indicating their potential key roles in biological processes related to energy metabolism, oxidative stress response, and muscle function. This network provides a preliminary interaction-based perspective on the molecular mechanisms underlying athletic performance in Grassland-Thoroughbreds.

## 4. Discussion

The Inner Mongolia region serves as an important genetic resource repository for Chinese horse breeds. With the gradual development of modern horse racing, developing local racehorse breeds has become increasingly important. In the horse breeding process, pedigrees are widely used to trace the origin of horses; however, they may lead to inaccurate estimations of inbreeding, increasing the risk of undesirable offspring. With the continuous advancement of sequencing technologies, whole-genome screening using SNP datasets has emerged as an alternative to pedigree records. This approach not only allows for more accurate assessment of inbreeding levels during the breeding process but also provides valuable information. Moreover, certain classes of ROH lengths can serve as indicators of pedigree and population history and help identify signatures of selection in the population genome during the breeding process.

Through long-term selective breeding, racehorse populations have gradually developed into specific superior breeds, with athletic traits often serving as key selection targets. ROH analysis enables the exploration of genetic diversity within populations, the evaluation and prediction of critical parameters of genomic structure, and the identification of structural regions that influence phenotypes through continuous homozygous segments. It can also be used to detect genes associated with athletic performance traits. In this study, the number and total length of short ROHs followed the order: F1 > F2 > CY > F3, whereas the number and total length of long ROHs exhibited the opposite trend. The F_ROH values showed the pattern: F1 < F2 < CY < F3. F1 contained more short ROH segments, indicating higher genetic diversity, lower relatedness, and low inbreeding levels. In contrast, F3 had the highest number of long ROHs and the highest inbreeding coefficient, likely due to repeated backcrossing with Thoroughbreds over successive generations, which increased the number of long ROHs. This suggests a greater degree of inbreeding and potential genetic drift in this group, leading to reduced genetic diversity. Although crossbreeding reduced relatedness, the effect was not significant. These findings suggest that recombination events occur during generational transmission and that ROH length is correlated with the number of ancestral generations.

To identify genomic regions with high-frequency ROHs and screen for candidate marker genes associated with athletic traits in different generations of the Grassland-Thoroughbred population, we conducted relevant analyses. Studies have shown that *FOXO1* is an important member of the FoxO family and plays a role in regulating energy homeostasis in muscle under catabolic conditions [22]. Exercise can induce the expression of *FOXO1* and enhance its activity, with its mRNA levels significantly upregulated after exercise [23,24]. *FOXO1* can bind to the promoter of the *PDK4* gene, regulating the expression of *PDK4* and lipoprotein lipase, thereby promoting glucose metabolism and fatty acid oxidation [25,26]. In Grassland-Thoroughbred horses, *FOXO1* may serve as a key regulatory factor in muscle stress adaptation and energy redistribution mechanisms during long-distance running and high-intensity training. Studies on the co-expression of exercise-regulated proteins in fast and slow muscle fibers have found that *NDUFB7*, a protein involved in oxidative phosphorylation, is upregulated after exercise [27]. In fast-running racehorse breeds, the SNP with the highest Fst ranking was found to involve *NDUFB7* [28], and this gene is directly regulated by *PPARA* in cancer models [29]. In Grassland-Thoroughbred horses, *NDUFB7* may be associated with the rate and efficiency of energy supply in muscle, thereby enhancing both short-term explosive power and sustained running capacity. *PPARA* plays a key role in exercise and training and is associated with physical performance in elite human endurance athletes [30]. *PRDX2* is a protein with antioxidant and molecular chaperone functions. Knockdown of *PRDX2* exacerbates muscle strength loss [31], highlighting its important role in regulating muscle function and oxidative stress in exercise-related studies. Research has shown that its expression fluctuates significantly after certain ultra-endurance exercises and can be upregulated through training [32,33]. Proteomic studies have found that endurance exercise can induce the expression of *PRDX2* [34]. In Grassland-Thoroughbred horses, *PRDX2* may work synergistically with *PPARA* to regulate energy metabolism and oxidative stress responses during high-intensity exercise. The high frequency of *PRDX2* and other genes within ROH regions may indicate their potential genetic contribution to the formation of superior athletic traits.

Neurobiological functions regulate racehorse performance in various aspects, including muscle coordination, sensory perception, and learning and memory. The *NTM* (neurotrimin) gene encodes a neural cell adhesion molecule, particularly associated with neurobiology and brain development [35]. Recent studies have shown that *NTM* influences behavioral traits in mice, with knockout mice exhibiting impaired learning ability [36]. In a genome-wide association study (GWAS) of Thoroughbreds, this gene locus showed a highly significant association with the number of races completed [37]. Among 125 genes identified as being under positive selection during horse domestication, *NTM* ranked ninth, suggesting potential overlap with the adaptive traits related to racing ability in Thoroughbreds [38]. Additionally, in efforts to identify fast-running racehorse breeds, *NTM* SNPs showed the strongest association with racing breeds (*p* = 7.49 × 10^−14^), with the highest allele frequency observed in Thoroughbreds [28]. Additionally, *OPCML* (opioid binding protein/cell adhesion molecule-like) was identified on chromosome 7. This gene shares functional similarity with *NTM*, as both encode members of the IgLON family of proteins, which are involved in regulating neural growth and synapse formation. The two genes may function cooperatively through complementary mechanisms [36]. Studies have shown that in the transcriptome analysis of equine skeletal muscle, *OPCML* exhibits significantly differential expression after training. Both *NTM* and *OPCML* are considered important predictive genes for racing participation in the genomic selection of Thoroughbreds [37,39]. *SLC4A10* plays a role in regulating neuronal cells and can significantly alter synaptic plasticity, learning ability, and physiological and behavioral traits such as neurodegeneration through pH-sensitive receptors and ion channels [40,41]. In *SLC4A10* knockout mice, ventricular volume is reduced and cerebrospinal fluid decreases [42]. Genetic variations in *SLC4A10* are associated with cognitive functions and other traits [43]. These genes (*NTM*, *OPCML*, *SLC4A10*) play important roles in neurobiology, not only contributing to enhanced athletic performance and rapid adaptation to training but also potentially aiding Grassland-Thoroughbred horses in quickly responding on the racetrack, managing complex race conditions, and recovering efficiently. The regulatory effects of these genes are crucial for the athletic ability and racing performance of Grassland-Thoroughbreds, and they can serve as potential genetic markers for screening in gene selection and molecular breeding processes.

Mitochondria are the primary energy generators within cells, playing a crucial role in supplying energy during equine exercise, as well as facilitating physical recovery and antioxidative processes. *ACAD8*, the eighth member of the acyl-CoA dehydrogenase family, functions as a dehydrogenase directly involved in mitochondrial oxidative metabolism. The rate of fatty acid β-oxidation is markedly elevated in football trainers, which correlates strongly with increased *ACAD8* expression levels [44]. In horses, the *ACAD8* gene is thought to influence muscle tissue architecture, and its single nucleotide polymorphisms (SNPs) have been utilized in cattle as genetic markers to enhance growth rates and meat quality traits [45]. *SLC25A15* (solute carrier family 25 member 15), as a key transporter protein located in the mitochondrial inner membrane, regulates the metabolism of ornithine and arginine [46], thereby promoting lipolysis and enhancing energy metabolism efficiency [47] while also maintaining a stable energy supply to the brain [48,49]. These candidate genes provide compelling evidence for the adaptive mechanisms underlying athletic performance in Grassland-Thoroughbreds. *ACAD8* and *SLC25A15* likely play pivotal roles in energy metabolism and exercise capacity in Grassland-Thoroughbreds, enhancing endurance, augmenting athletic performance, and promoting recovery efficiency, thereby enabling these horses to maximize their potential under high-intensity exercise conditions.

With the rapid development of modern breeding methods and horse racing competitions, the Grassland-Thoroughbred, as an important locally bred racing breed, has formed a unique genetic background and adaptive traits through long-term natural selection and artificial domestication. It also carries many valuable and excellent traits that are of great significance for enhancing the competitiveness of China’s horse industry. Genetic research and conservation of the Grassland-Thoroughbred can provide abundant genetic resources for breeding. This study reveals the trends of inbreeding accumulation, changes in genetic structure, and evolutionary trajectories across different generational groups through ROH analysis. Based on these findings, breeding programs can be reasonably designed to avoid the adverse effects caused by excessive inbreeding. Additionally, potential genetic segments can be identified within ROH islands, providing a basis for subsequent functional validation and molecular marker development, thereby guiding targeted breeding efforts.

This study has several limitations. Although ROHs have advantages in assessing inbreeding and autozygosity, their detection results are influenced by factors such as marker density, marker selection, and individual differences in recombination rates, which impose certain limitations. The relatively small sample size may affect the statistical power of the results and the generalizability of the conclusions. Future studies should increase the sample size to enhance the representativeness and reliability of the research. Secondly, although efforts were made to exclude close relatives through population structure analysis, potential population stratification may still exist, which could introduce bias into the genetic association analysis. The lack of detailed athletic performance phenotype data means that the genetic basis underlying phenotypic differences in horses requires further in-depth exploration. Through whole-genome resequencing and association analysis, several candidate genes, including *FOXO1*, *ACAD8*, and *PRDX2*, were identified and speculated to be related to athletic traits in this breed. However, due to the absence of experimental validation of these genes’ functions, the current relationship between them and athletic performance remains speculative. Given that gene functions may differ across species, future research should focus on systematic molecular biology experiments and animal model validations to further clarify the specific regulatory mechanisms of these candidate genes in equine athletic ability. By integrating these molecular markers into precise selection and genetic improvement programs, it is expected to enhance the athletic performance and adaptability of the Grassland-Thoroughbred, thereby promoting its sustained competitiveness in both domestic and international racing industries. This study provides valuable foundational data and preliminary insights into the molecular mechanisms related to athletic performance while also offering potential genetic markers for optimizing breeding strategies.

## 5. Conclusions

This study conducted a comprehensive analysis of whole-genome sequencing data across multiple generations of the Grassland-Thoroughbred population. Clear differentiation was observed among different generations, except between CY and F3. The trends in runs of homozygosity (ROH) segments and inbreeding coefficients reflect a gradual decline in genetic diversity with increasing generations. Notably, ROH island analysis identified multiple candidate genes—such as *ACAD8*, *OPCML*, *PRDX2*, *NTM*, *NDUFB7*, *SLC25A15*, *FOXO1*, and *SLC4A10*—that may be involved in key physiological processes including muscle function, energy metabolism, and cognitive ability, all of which have important impacts on athletic performance. The findings deepen our understanding of the genetic basis of athletic traits in the Grassland-Thoroughbred and provide important targets for future breeding strategies. Overall, this study lays a solid foundation for the genetic improvement and optimization of this breed, contributing to its sustainable development and enhancement of competitive advantages.

## Figures and Tables

**Table 1:** Distribution of ROH islands throughout the genome.

Chr	Start Position	End Position	SNPs	Genes	Gene Symbol
3	46675626	47346334	221	1	*GRID2*
7	41513334	47138251	7254	83	*NTM*, *OPCML*, *SPATA19*, *IGSF9B*, *JAM3*, *NCAPD3*, *VPS26B*, *ACAD8*, *THYN1*, *LOC100072895*, *LOC100072910*, *B3GAT1*, *LOC100064396*, *LOC111774264*, *LOC102149984*, *LOC111774398*, *LOC100064454*, *LOC100064510*, *LOC100064538*, *ZNF333*, *ADGRE3*, *CLEC17A*, *NDUFB7*, *TECR*, *DNAJB1*, *GIPC1*, *PTGER1*, *PKN1*, *DDX39A*, *ADGRE5*, *ADGRL1*, *PRKACA*, *SAMD1*, *C7H19orf67*, *MISP3*, *PALM3*, *IL27RA*, *RLN3*, *RFX1*, *DCAF15*, *PODNL1*, *CC2D1A*, *C7H19orf57*, *NANOS3*, *ZSWIM4*, *C7H19orf53*, *MRI1*, *CCDC130*, *CACNA1A*, *IER2*, *STX10*, *NACC1*, *TRMT1*, *LYL1*, *NFIX*, *DAND5*, *GADD45GIP1*, *RAD23A*, *CALR*, *FARSA*, *SYCE2*, *GCDH*, *KLF1*, *DNASE2*, *MAST1*, *RTBDN*, *RNASEH2A*, *PRDX2*, *JUNB*, *HOOK2*, *BEST2*, *ASNA1*, *TRIR*, *TNPO2*, *FBXW9*, *GNG14*, *DHPS*, *WDR83*, *WDR83OS*, *MAN2B1*, *LOC100064480*, *LOC100064534*, *LOC100064626*
7	48278064	48878342	508	8	*LOC102147654*, *LOC102149586*, *LOC102149528*, *LOC100629361*, *LOC100066109*, *LOC100629395*, *LOC100066251*, *LOC102147741*
7	50444481	50450375	4	0	
11	31313080	31545459	256	2	*NOG*, *C11H17orf67*
17	18312535	18944395	1180	3	*FOXO1*, *MRPS31*, *SLC25A15*
18	41668278	42417738	526	6	*TANK*, *LOC111768910*, *PSMD14*, *TBR1*, *SLC4A10*, *DPP4*
18	49354856	50138296	771	4	*SSB*, *METTL5*, *UBR3*, *MYO3B*
21	29890004	30513697	7	13	*LOC111769792*, *LOC111769791*, *LOC111769793*, *CAPSL*, *LOC111769795*, *LOC111769798*, *LOC111769796*, *LOC111769800*, *LOC111769799*, *LOC111769801*, *LOC111769804*, *LOC111769802*, *SPEF2*
28	58964	685343	1237	0	

## Data Availability

Sequence data that support the findings of this study have been deposited in the National Center for Biotechnology Information with the primary accession code PRJNA1287091.

## Supplementary Materials

The following supporting information can be downloaded at: https://www.mdpi.com/article/10.3390/ani15142068/s1. Supplementary Table S1: DNA quality; Supplementary Table S2: The statistical table of sequencing results for each sample; Supplementary Table S3: ANNOVAR (https://annovar.openbioinformatics.org/en/latest/user-guide/download/, accessed on 12 June 2023) annotation results; Supplementary Table S4: Functional enrichment results of candidate genes; Supplementary Figure S1: Breeding route of the Grassland-Thoroughbreds; Supplementary Figure S2: The distribution of SNPs on different chromosomes.
