# Supplementary material for: Analysis of ROH Characteristics Across Generations in Grassland-Thoroughbred Horses and Identification of Loci Associated with Athletic Traits
Source: Animals (Basel). 2025 Jul 13;15(14):2068. doi: 10.3390/ani15142068 (PMC12291906; doi:10.3390/ani15142068)

Author 1

Name: Wenqi Ding

Affiliation: Inner Mongolia Agricultural University

Email: dingwenqi0331@gmail.com

| Education Background: | | |
| --- | --- | --- |
| University name: Inner Mongolia Agricultural University | College name: College of Animal Science | Educational level: Master |

Signature:


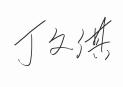


Author 2

Name: Wendian Gong

Affiliation: Inner Mongolia Agricultural University

Email：gongwendian1996@outlook.com

| Education Background: | | |
| --- | --- | --- |
| University name: Inner Mongolia Agricultural University | College name: College of Animal Science | Educational level: Doctor |

Signature:


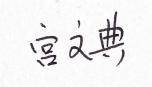


Author 3

Name: Tugeqin Bou

Affiliation: Inner Mongolia Agricultural University

Email: tvgqin@gmail.com

Signature:


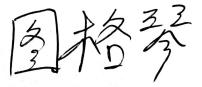


Author 4

Name: Lin Shi

Affiliation: Inner Mongolia Agricultural University

Email: 19832607527@163.com

| Education Background: | | |
| --- | --- | --- |
| University name: Inner Mongolia Agricultural University | College name: College of Animal Science | Educational level：Bachelor |

Signature:


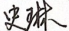


Author 5

Name: Yanan Lin

Affiliation: Inner Mongolia Agricultural University

Email: linyanan@emails.imau.edu.cn

| Education Background: | | |
| --- | --- | --- |
| University name: Inner Mongolia Agricultural University | College name: College of Animal Science | Educational level: Doctor |

Signature: .


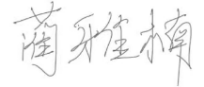


Author 6

Name: Xiaoyuan Shi

Affiliation: Inner Mongolia Agricultural University

Email: xiaoyuans2021@163.com

| Education Background: | | |
| --- | --- | --- |
| University name: Inner Mongolia University | College name: College of Life Science | Educational level：Bachelor |

Signature:


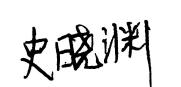


Author 7

Name: Zheng Li

Affiliation: Inner Mongolia Agricultural University

Email: lzheng0511@sina.com

| Education Background: | | |
| --- | --- | --- |
| University name: Inner Mongolia University | College name: College of Life Science | Educational level：Bachelor |

Signature:


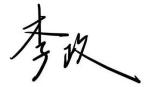


Author 8

Name: Huize Wu

Affiliation: Inner Mongolia Agricultural University

Email: whz020419@163.com

| Education Background: | | |
| --- | --- | --- |
| University name: Inner Mongolia University | College name: College of Life Science | Educational level：Bachelor |

Signature:


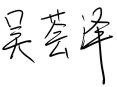


Author 9

Name: Manglai DUGARJAVIIN

Affiliation: Inner Mongolia Agricultural University

Email: dmanglai@163.com

Educational level: Doctor

Signature:


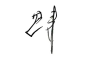


Author 10

Name: Dongyi Bai

Affiliation: Inner Mongolia Agricultural University

Email: baidongyi1983@163.com

Educational level: Doctor

Signature:


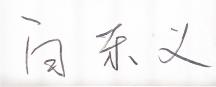


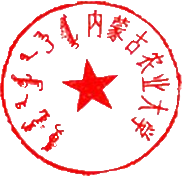

Supplement: Supplementary file 1 [file animals-15-02068-s001.zip › Author's CV.docx]
