# Supplementary material for: Analysis of ROH Characteristics Across Generations in Grassland-Thoroughbred Horses and Identification of Loci Associated with Athletic Traits
Source: Animals (Basel). 2025 Jul 13;15(14):2068. doi: 10.3390/ani15142068 (PMC12291906; doi:10.3390/ani15142068)

同意书

我，内蒙古草原纯血马培育有限公司同意参与关于内蒙古农业大学马属动物及其马匹运动性状相关研究项目。

我已阅读并理解提供给我的信息表，其中包括：

·本研究项目由内蒙古农业大学马属动物研究中心的研究人员开展，目的是收集关于关于蒙古马、锡林郭勒马、草原纯血马运动性状等方面的定性数据，数据将用于改进育种策略以及分析相关基因对马匹运动表现的影响。

·我理解研究结果可能会在本文中报告。

·我已被告知该项目的性质。

·我有权可以随时退出研究，且不会因此受到任何不利后果。

·我理解，在本研究过程中收集的关于我的任何身份信息都是保密的，未经我的书面许可不会使用或公开。

· 我理解，文章的发表不会为我带来任何财务收益或补偿。

我理解，如果我对这项研究有任何疑问、投诉或疑虑，我可以随时联系该项目的首席研究员，或可以联系以下联系人：

研究负责人：白东义

电子邮箱：baidongyi1983@163.com

签名：


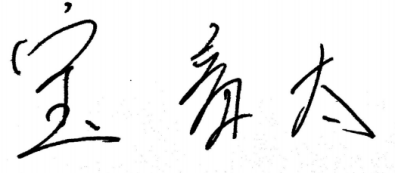

Supplement: Supplementary file 1 [file animals-15-02068-s001.zip › blank consent form 1.docx]
