# Supplementary material for: Analysis of ROH Characteristics Across Generations in Grassland-Thoroughbred Horses and Identification of Loci Associated with Athletic Traits
Source: Animals (Basel). 2025 Jul 13;15(14):2068. doi: 10.3390/ani15142068 (PMC12291906; doi:10.3390/ani15142068)
Supplement: Supplementary file 1 [file animals-15-02068-s001.zip › Inner Mongolia Agricultural University Biomedical Research Ethics Approval.pdf]

内蒙古農業大學
ᠦᠪᠦᠷ ᠮᠣᠩᠭᠣᠯ ᠤᠨ ᠬᠥᠳᠡᠭᠡ ᠠᠵᠤ ᠠᠬᠤᠢ ᠶᠢᠨ ᠶᠡᠬᠡ ᠰᠤᠷᠭᠠᠭᠤᠯᠢ

内蒙古农业大学生物医学科研伦理审批件

编号(No)：NND2023085

我校教师芒来申请的 2023 年国家自然科学基金项目《基于多组学技术对蒙古马耐力特性形成的分子机制解析》,经内蒙古农业大学实验动物福利与伦理委员会的审核，符合伦理原则，同意申报国家自然科学基金项目。

内蒙古农业大学实验动物福利与伦理委员会

内蒙古农业大学科技处（代章）

2023 年 3 月 10 日

内蒙古农业大学 科技处 1501051007705б

地址：中国•内蒙古•呼和浩特市昭乌达路306号 邮编：010018
电话：0471-4309281 0471-4309264 传真：0471-4301530
