# Supplementary material for: Analysis of ROH Characteristics Across Generations in Grassland-Thoroughbred Horses and Identification of Loci Associated with Athletic Traits
Source: Animals (Basel). 2025 Jul 13;15(14):2068. doi: 10.3390/ani15142068 (PMC12291906; doi:10.3390/ani15142068)
Supplement: Supplementary file 1 [file animals-15-02068-s001.zip › MDPI-Authorship-Form.pdf]

Multidisciplinary
Digital Publishing
Institute

MDPI AG
Postfach
CH–4057 Basel
Switzerland

Tel. +41 61 683 77 34
Fax +41 61 302 89 18
E-Mail: rordorf@mdpi.com
www.mdpi.com

Authorship Contribution Form

(Must be Completed and Signed by ALL Authors)

MDPI follows the International Committee of Medical Journal Editors (ICMJE) guidelines which state that, in order to qualify for authorship of a manuscript, the following criteria should be observed:

- Substantial contributions to the conception or design of the work; or the acquisition, analysis, or interpretation of data for the work; AND
- Drafting the work or revising it critically for important intellectual content; AND
- Final approval of the version to be published; AND
- Agreement to be accountable for all aspects of the work in ensuring that questions related to the accuracy or integrity of any part of the work are appropriately investigated and resolved.

MDPI journals require that all authors indicate their specific contributions to the preparation of this manuscript by completing this form.

Journal Name:	animals
Manuscript ID (if known):	
Manuscript Title:	Analysis of ROH Characteristics Across Generations in Grassland Thoroughbred Horses and Identification of Loci Associated with Athletic Traits
Authors:	Wenqi Ding, Wendian Gong, Tugeqin Bou, Lin Shi, Yanan Lin, Xiuaoyuan Shi, Zheng Li, Huize Wu, Manglai Dugarjaviin
Corresponding Author(s):	Dongyi Bai

All authors of the paper should:

☐ confirm that all listed authors meet the criteria above and all who meet the four criteria are identified as authors

☐ confirm that the manuscript has been read and approved by all named authors

☐ confirm that the order of authors listed in the manuscript has been approved by all named authors

Please list all the manuscript authors and their contribution in the Contribution Table below. The corresponding author will be responsible to include a summary statement in the text of the manuscript in a separate section "Author Contributions", that reflects what is recorded in the following table.

List ALL AUTHORS in the same order as the manusript. (If additional author and signature fields are needed, please duplicate this form as needed):

**Name**	**Contribution (please refer to CrediT in Appendix)**	**Signature**	**Date**
Wenqi Ding	Conceptualization; Visualization; Writing - original draft; Writing - review & editing;		9 June 2025
Wendian Gong	Formal analysis; writing—original draft preparation; writing—review and editing		9 June 2025
Tugeqin Bou	Software; Investigation;		9 June 2025
Lin Shi	Software; Writing - review & editing		9 June 2025
Yanan Lin	Formal analysis; software		9 June 2025
Xiuaoyuan Shi	Writing - original draft; visualization		9 June 2025
Zheng Li	Software; visualization		9 June 2025
Huize Wu	Investigation; software		9 June 2025
Manglai Dugarjaviin	Resources; Project administration; Funding acquisition		9 June 2025
Dongyi Bai	Conceptualization; Resources; Project administration; Funding acquisition		9 June 2025

内蒙古农业大学

Appendix

Contributor Roles Taxonomy (CRediT)

Conceptualization	Ideas; formulation or evolution of overarching research goals and aims.
Data curation	Management activities to annotate (produce metadata), scrub data and maintain research data (including software code, where it is necessary for interpreting the data itself) for initial use and later re-use.
Formal analysis	Application of statistical, mathematical, computational, or other formal techniques to analyze or synthesize study data.
Funding acquisition	Acquisition of the financial support for the project leading to this publication.
Investigation	Conducting a research and investigation process, specifically performing the experiments, or data/evidence collection.
Methodology	Development or design of methodology; creation of models.
Project administration	Management and coordination responsibility for the research activity planning and execution.
Resources	Provision of study materials, reagents, materials, patients, laboratory samples, animals, instrumentation, computing resources, or other analysis tools.
Software	Programming, software development; designing computer programs; implementation of the computer code and supporting algorithms; testing of existing code components.
Supervision	Oversight and leadership responsibility for the research activity planning and execution, including mentorship external to the core team.
Validation	Verification, whether as a part of the activity or separate, of the overall replication/reproducibility of results/experiments and other research outputs.
Visualization	Preparation, creation and/or presentation of the published work, specifically visualization/data presentation.
Writing - original draft	Preparation, creation and/or presentation of the published work, specifically writing the initial draft (including substantive translation).
Writing - review & editing	Preparation, creation and/or presentation of the published work by those from the original research group, specifically critical review, commentary or revision – including pre- or post-publication stages.
